# The Recurrence of Painful Neuromas of the Limbs Following TMR

**DOI:** 10.3390/jcm14041078

**Published:** 2025-02-08

**Authors:** Alessandro Crosio, Elisa Rosanda, Francesca Latini, Alice Clemente, Francesco Maria Locatelli, Mauro Magnani, Letizia Marenghi, Pierluigi Tos

**Affiliations:** 1 Reconstructive Microsurgery Unit, Department of Orthopedics & Traumatology, AOU Città della Salute e della Scienza di Torino, 10126 Torino, Italy; alessandro.crosio@unito.it; 2 Department of Hand Surgery and Reconstructive Microsurgery, ASST Gaetano Pini-CTO, Piazza A. Ferrari 2, 20122 Milano, Italy; 3 UO Chirurgia della Mano, Ospedale Multimedica, 20123 Milano, Italy; 4 UOC Ortopedia, Traumatologia e Chirurgia della Mano, Ospedale Belcolle, 01100 Viterbo, Italy

**Keywords:** painful neuroma, TMR, chronic pain, nerve injury

## Abstract

**Background/Objectives:** Neuropathic pain associated with neuromas is a complex clinical problem to treat. Targeted Muscle Reinnervation (TMR) has been demonstrated to treat pain both as a prophylactic procedure in amputated patients and in patients affected by painful neuromas. It is not clear what its role could be in chronic situations: the literature reports amazing results but also unsuccessful pain relief. **Methods**: A retrospective analysis was conducted on patients treated with TMR for long-lasting painful neuromas in the upper and lower limbs. Following a clinical and instrumental diagnosis, all patients responded positively to a local anesthetic block. During follow-up visits, the NRS and DN4 questionnaires were used to assess improvement in pain. **Results**: Three patients were included in this study. TMR was performed 45 months after trauma. Two TMRs involved nerves of the upper extremity, in one case, the tibial nerve. The recipient muscles were the second lumbricalis, pronator quadratus, and flexor digitorum longus of the foot. After surgery, pain decreased for 3 months, but patients experienced a relapse that returned to levels close to the pre-operative period. The types of pain, as reported in DN4 questionnaire, changed slightly compared to those in the pre-surgical period. Follow-up ranged between 12 and 19 months. **Conclusions**: This small series collected the results of TMR in patients affected by long-lasting symptomatic neuromas in the upper and lower extremities. Despite what is published in other series, this procedure reduced pain for up to 6 months. At final follow-up, the type of pain changed slightly as reported in the DN4 questionnaire, and pain scores reduced by just one point as shown by the NRS. Our experience suggests that TMR might have a slight effect on long-lasting painful neuromas and in these cases, only short-term pain relief could be expected. This suggests using TMR as close as possible to the trauma in order to increase the chances of relieving pain.

## 1. Introduction

Neuromas typically develop at the site of nerve injury, which may result from a cut, stretch, crush, or other types of nerve damage [1]. Following nerve injury, the nerve tries to regenerate; however, this process may result in the formation of a neuroma, a mass of nerve fibers that fails to reconnect properly. Neuromas frequently cause pain, which can be sharp, burning, or resemble an electric shock, along with tingling or numbness and sensitivity to touch [2].

It is therefore understandable how these symptoms affect a person’s quality of life and often require medical and surgical intervention.

The diagnosis of a neuroma is primarily clinical and is suspected when symptoms persist beyond initial recovery.

Key diagnostic methods include the Tinel sign, elicited by percussing the nerve’s course, and noninvasive imaging like ultrasonography and MRI for confirmation and detailed assessment.

Diagnostic blocks with local anesthetic can be used to localize neuropathic pain; however, their clinical significance in guiding treatment decisions is still controversial. If a block does not relieve pain, it suggests that surgery may not be helpful; however, if the nerve responds to anesthetic, surgery may not necessarily be decisive [3].

To date, treatment and prevention of painful neuromas remain a complex challenge. Currently, there is no universally agreed-upon approach for the optimal management of painful neuromas. Consequently, a wide range of approaches to treat neuroma pain have been described, encompassing pharmacological, psychological, and physical interventions.

Generally, surgery depends on whether a distal stump is present and whether its function is crucial.

If a distal nerve is unavailable, regenerative peripheral nerve interfaces (RPNIs) and targeted muscle reinnervation (TMR) are two active techniques that seem to offer the most promise in preventing and treating terminal neuroma formation, even if standardization in reporting surgical techniques, outcomes, and confounding factors is needed in future studies. Other procedures proposed in such situations include relocation of the proximal stump into muscle, nerve capping, and centro-central anastomosis (CCA). The latter are defined as passive procedures, while the former are supposed to be active procedures, giving axons the possibility to reach a distal target [4].

TMR was initially developed by Kuiken et al. to enhance myoelectric prosthesis function for amputees [5,6].

Beyond prosthetic improvement, TMR has become the method of choice to treat painful neuromas post-amputation, showing promise in both amputees and non-amputees. This technique involves connecting cut sensory or mixed nerves to the distal end of an intact muscle motor nerve using t-t neurorrhaphy, providing a new target for nerve growth and potentially reducing neuroma formation and pain [7].

To limit regeneration time, it is strongly recommended to shorten the distal target motor nerve stump as much as possible while preserving a tension-free nerve coaptation. The neurorrhaphies are performed within 1–2 mm of where the recipient motor nerves enter the recipient muscles [8]. Several studies highlight TMR’s effectiveness in preventing neuroma formation and recurring pain, with significant pain relief reported in patient series. TMR has been adapted to various applications, including the treatment of chronic nerve pain and prophylactic use during nerve transection, such as in ray amputations. Technical challenges include identifying suitable motor end points, ensuring tension-free nerve coaptation, and managing size mismatches at the coaptation site.

In the last few years, TMR has been proposed as an alternative method to treat painful neuromas. Its efficacy is demonstrated more for prevention than for the treatment of painful neuromas. In this field, TMR is still under evaluation to understand its true effectiveness.

The objective of this study was to describe our experience with TMR in three patients affected by long-lasting painful neuromas of distal segments of the body and reports its efficacy on pain resolution.

## 2. Materials and Methods

We examined patients affected by chronic painful neuromas who underwent TMR in the Reconstructive Microsurgery Department of ASST Gaetano Pini—CTO in Milan between 2020 and 2023. The efficacy of the surgical procedure was evaluated by NRS and DN4 questionnaires during serial follow-up visits. The most recent follow-up was in November 2024.

All patients were evaluated with a specific nerve ultrasound before surgery to confirm the presence of neuromas. Furthermore, all patients underwent a local anesthetic block with lidocaine proximal to the neuromas, which confirmed disappearance of pain.

After exposure, the neuroma(s) was/were transected back to healthy nerve tissue, confirmed by punctate bleeding and absence of macroscopic scar areas in the nerve section. Nerve trunks were then sutured using a 9-0 nylon suture and optical magnification devices. In some cases, a difference in caliber was observed, as it is well documented in the TMR procedure.

All patients gave written consent for this publication, and a copy of the consents is kept at the treating institution.

## 3. Results

Three patients were included.

Table 1 summarizes the physiological characteristics and medical history of each patient.

### 3.1. Patient 1

The patient was affected by a tear amputation of the thumb apex that was complicated by painful terminal neuromas of the sensory volar nerves, initially treated with second phalanx amputation, neuroma resection, and CCA of volar digital nerves. It was then treated due to a double crush syndrome of the median nerve at the carpal tunnel and flexor superficialis muscle arch. The patient subsequently developed a painful neuropathy of the sensory radial nerve due to the presence of small terminal neuromas at the dorsum of the thumb. According to the patient’s preferences, TMR was performed between the radial sensory nerve (RSN) and the motor branch of the pronator quadratus at the wrist level.

### 3.2. Patient 2

**Figure 1:**
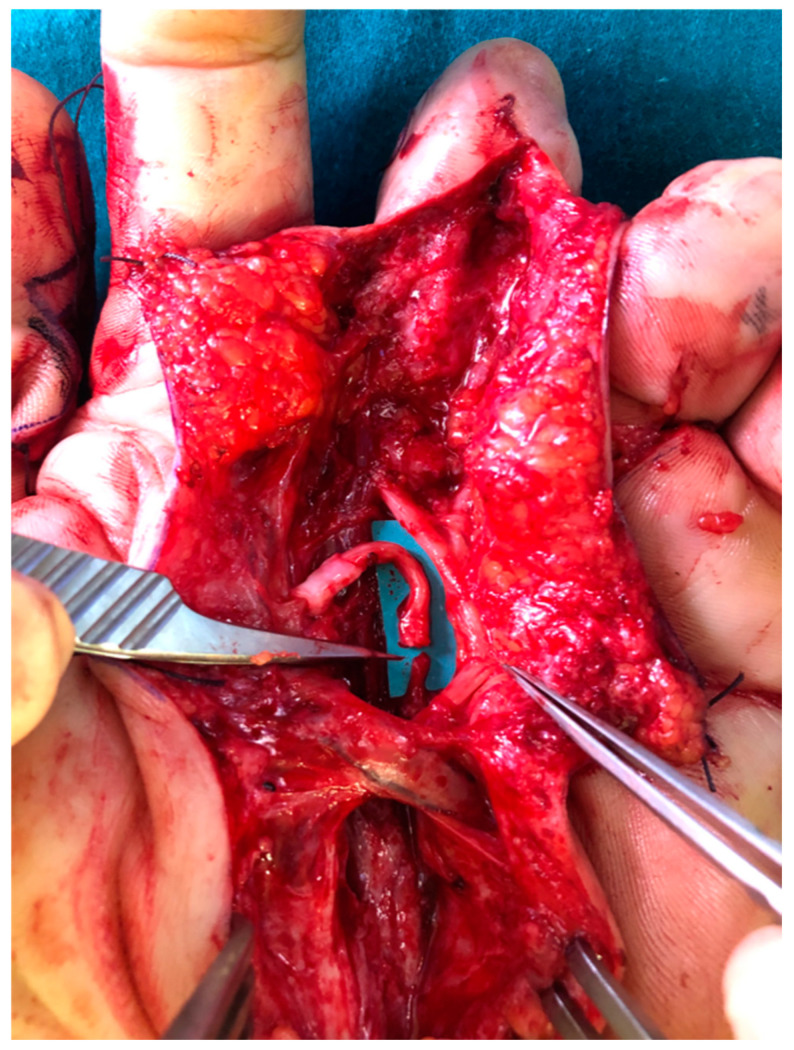
The ulnar digital nerve of the middle finger is sutured to the second lumbricalis motor nerve, flipped distally to allow direct suture beyond the palmar arch.

This patient experienced a crush injury to the middle and ring fingers at the proximal interphalangeal joint level, and the thumb was subamputated at the interphalangeal joint. The long fingers were finally amputated two months after the trauma. He developed painful neuromas, which were resected, and the nerve stumps were covered with nerve caps more than two years after the trauma. As a second recurrence of painful neuromas arose, the TMR procedure was proposed to prevent further recurrences in the stumps of the middle finger. The recipient nerve was the motor branch of the second lumbricalis muscle, as shown in Figure 1. Additional procedures performed included centro-central union for the collateral digital nerves of the ring finger and direct muscle neurotization of the ulnar collateral nerve of the middle finger. During follow-up, 12 months after TMR, due to referred pain, high-resolution ultrasonography showed a recurrence of neuromatous enlargement of the collateral digital nerve at the site of the previous TMR, confirming the relapse of painful neuroma.

### 3.3. Patient 3

This patient’s right leg was injured by a motor hoe, severing the tibial nerve, which was not initially recognized in the emergency department. Due to a painful neuroma of the tibial nerve, it was reconstructed one year later when she was referred to our institution. Two years after nerve reconstruction, there were no signs of nerve recovery, and a painful neuroma appeared at the proximal suture of the graft.

**Figure 2:**
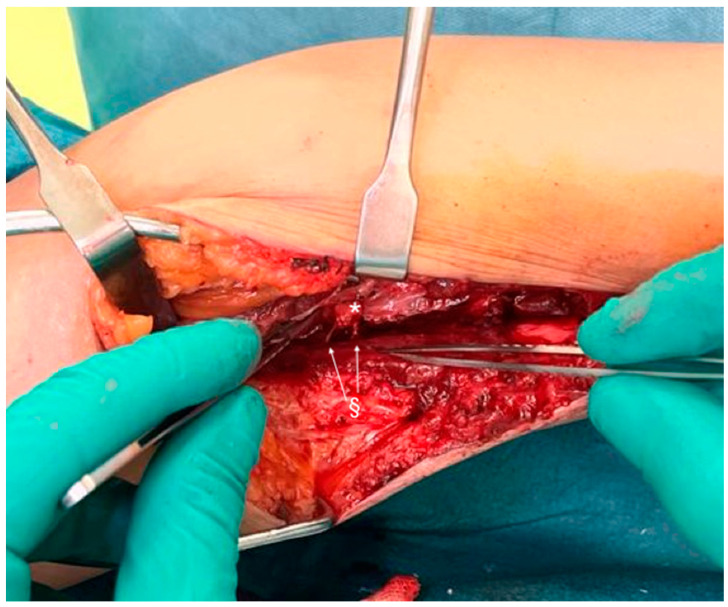
The proximal stump of the tibial nerve (*) is sutured to the two motor branches of the flexor digitorum longus of the foot (§).

In an attempt to alleviate her pain, a TMR was planned, connecting the tibial nerve to the motor branch of the flexor digitorum longus of the foot, as reported in Figure 2.

Table 2 reports the type of TMR procedure, the time between trauma and TMR, and outcomes in terms of pain reduction. TMR was performed at a mean of 45 months after the trauma. All patients had undergone at least one previous procedure in which terminal neuromas or neuromas in continuity had been resected. The patients were followed up for a minimum of 12 months, with the longest follow-up period being 19 months. Pain decreased after surgery for a mean of 3 months, then returned to levels comparable to those experienced preoperatively. The longest pain-free period was 6 months. TMRs were directed to the second lumbrical muscles, the flexor digitorum longus of the foot, and the pronator quadratus.

## 4. Discussion

The superiority of any one surgical treatment over another is largely debated for the treatment of painful neuromas of the limbs.

Classical techniques, which include neuroma excision, nerve capping, excision with transposition into bone or muscle, and centro-central union, have been defined as “passive procedures”, since axons are not directed towards a distal target such as motor end plates or sensory receptors [4].

One of our patients was treated with a nerve cap. A recent multicenter study [9] showed the efficacy of these devices in preventing neuroma relapse in a large sample. Additionally, an extensive literature review [10] further reported its efficacy despite the type of capping used. This review, however, reported a small number of patients with persistent relapsing pain. Why this happened was not investigated, and it is unclear what conditions contribute to the failure of this treatment.

Conversely, based on the concept that a nerve needs “somewhere to go and something to do”, the so-called active procedures are increasingly used in this field. They have been summarized in a recent paper as those that allow the nerve to reinnervate “something”. The active procedures, when a distal stump is not available, are TMR and RPNI. Primarily utilized in lower limb procedures, TMR has, in recent years, increasingly been applied to upper limb surgeries. Both of these procedures should provide a target for axonal regeneration to limit excessive nerve growth without causing significant donor site morbidity [11].

Regenerative Peripheral Nerve Interface is an alternative procedure to TMR, which has shown comparable results and can be used to treat painful neuromas as well [12].

Despite the absence of a randomized controlled trial comparing “passive” procedures among them, it is believed by experts that there is no superiority of one of these procedures over another; furthermore, it is increasingly believed that “active” procedures are superior compared to the classic ones and should always be preferred over the others [7].

TMR and RPNI have demonstrated their efficacy over the years and showed the highest success rate when performed immediately after amputation or soon after a painful neuroma is diagnosed.

The systematic review by Berger et al., which assesses TMR efficacy in lower extremity amputations, highlights its role in reducing phantom limb pain and residual limb pain with minimal complications [13].

This is echoed in studies by Alexander et al., Bowen et al., and Valerio et al., which not only confirm TMR effectiveness in alleviating pain, but also in reducing opioid dependency post-surgery [14,15,16].

Furthermore, the comparison between acute and delayed TMR showcases the importance of timely intervention. Research conducted by Reid et al. and Goodyear et al. demonstrates that implementing TMR shortly after amputation—within one month—can markedly enhance pain management and diminish the risk of neuroma development. This report highlights the advantages of integrating TMR into the initial stages of post-amputation treatment. However, it is noteworthy that in one of these studies, follow-up periods, such as 10 months in one study, may not be enough to assure that recurrence does not occur. This aspect suggests the need for longer-term evaluations to fully understand the enduring impacts of TMR [17,18].

Furthermore, Daugherty et al. highlight the successful use of TMR in treating recurrent neuropathic pain in the hand following finger amputation [19]. This development has paved the way for innovative uses and broader implementation of the technique, both in preventing and addressing neuropathic pain associated with digital neuromas.

Most of the recent literature investigates the use of TMR soon after symptoms appear, but it is unclear, and few reports have been published on its use when treating long-lasting painful neuromas.

We presented here three cases of painful neuromas of the limbs that arose over a period of more than two years. In all of our patients, TMR may offer some benefits, particularly in terms of associated symptoms other than pain, as indicated by the DN4 questionnaire. However, the overall level of pain reported remains high, and pain recurrence within a few months post-surgery can be anticipated, as it can be associated with central synaptic changes, perpetuating pain even without active pain fiber stimulation. This phenomenon might be more pronounced in areas like the volar digital nerves, which have extensive cortical representation compared to more proximal body areas. Indeed, research suggests that volar digital nerves are more susceptible to developing symptomatic neuromas than dorsal nerves [20]. Cortical changes associated with time from trauma can be considered one of the main reasons leading to the failure of TMR in our series. Changes in the spinal cord may also maintain pain even when the distal neuroma is resected. Despite not taking neuroma morphology into account, some anatomical characteristics can be associated with painful neuroma and TMR failure [21].

Brain and spinal cord plasticity associated with cortical representation of sensory areas can be the basis for the failure of our treatment. The mechanism through which TMR works is not completely understood. Pre-clinical studies have tried to clarify it, but without definitive success. Senger and colleagues, in 2023, published a pre-clinical paper that showed the reinnervation of motor plates through TMR and, moreover, demonstrated a significant decrease in the expression of proteins traditionally associated with nociception (NPY, CGRP, and TRPV1) and regeneration (pCREB and ATF-3) when compared with controls, suggesting these techniques modulate pain at the level of the cell body [22]. This paper analyzes what happens after neuroma resection and TMR, but only in a short-time setting. The treatment was performed 6 weeks after nerve transaction. This is dissimilar to what happened in our cases, where numerous months passed after neuroma induction, allowing the pain to be corticalized.

In recent literature, our paper is not the only one describing the failure of TMR. In 2021, Felder [23] reported two cases in which neuromas appeared and became painful in amputees who had undergone TMR as first-line treatment. In his paper, possible reasons for failure were discussed, such as excessive distance to target muscles, nerve kinking, size mismatch, and incorrect selection of the receiving muscle. Looking back at our patients, it is difficult to clearly allocate failure into one of these categories, especially since no revision surgery was performed. To avoid collateral sprouting in the surrounding tissue, either vein coverage of the coaptation site or muscle wrapping of the suture site was performed, in patients 2 and 3, respectively. Despite this, pain and neuromas relapsed, adding new aspects to be discussed regarding TMR.

Finkelstein et al., interestingly, studied patients undergoing upper limb amputation and TMR reconstruction through ultrasound. In one case, successful reinnervation was indicated by the continuity of nerve fascicles and their integration into the target muscle, showcasing the procedure’s effectiveness. In contrast, another patient’s ultrasound revealed discontinuity in nerve fascicles, neuroma formation, and muscle atrophy across all sites of nerve transfer, pointing to a less successful outcome and compromised functional recovery [24]. This report is similar to what was observed in patient 2, in whom a novel neuroma was found one year after TMR, when the patient began to complain of pain.

From a clinical point of view, an interesting aspect related to the clinical management of these patients is the use of pre-operative local anesthetic block. All our patients responded positively to the local anesthetic. This response should be considered a prerequisite for surgery. Despite these positive initial responses, the outcomes were not favorable. We consider this a key factor in the treatment of painful neuromas, although most of the papers addressing this problem do not.

Furthermore, it is very important to appropriately select an expandable recipient muscle. van Opijnen et al. reported the treatment of a recurrent traumatic neuroma of the posterior sural nerve. After two unsuccessful resections, TMR was employed, connecting the sural nerve to the lateral gastrocnemius muscle, leading to significant pain reduction over time without compromising plantar flexion strength. However, subsequent imaging showed muscle atrophy, highlighting the complexities of postoperative recovery [25]. This instrumental finding shows one of the main drawbacks of TMR: sacrificing a functioning muscle. No other report in the literature demonstrated this aspect so clearly. We considered this aspect in our practice. This should be useful, especially in the hand. When the proximal interphalangeal joint is present, one should consider the importance of lumbricalis and interossei muscles in finger extension. In our case, the patient had the PIP joint previously amputated, allowing the lumbricalis (or interossei) to be expandable.

Painful neuromas of our series involved different nerves. Depending on the type of nerve treated, different expandable muscles can be chosen. When considering the treatment of SRN neuropathy, the most accessible nerve to connect to the sensory nerve is the pronator quadratus branch from the anterior interosseous nerve. Alternatively, more proximal TMR has been proposed as an approach other than the pronator quadratus. Some authors have, in fact, proposed to go more proximal with nerve resection, thinking that the presence of a more proximal site of compression could be the reason for the non-resolution of pain. For this reason, more proximal TMR has been proposed using the branches of the brachioradialis and the extensor carpi radialis brevis muscles or the supinator motor branches [26,27]. This approach has the advantage of bypassing any proximal branching of the SRN that could be preserved by connecting the distal third to the AIN. Despite this, as noted in Ferrin’s paper, pain can persist, as observed in patient 3.

## 5. Conclusions

This small case series is the first in the literature to report four TMR procedures with disappointing outcomes. One of our patients experienced pain relief for 6 months before relapsing. The other patients reported the same level of pain as before, although the DN4 questionnaire indicated a change in the type of pain described. This implies that TMR for chronic, long-lasting painful neuromas affecting the hand and foot may not be successful. According to our research, TMR may help with pain management to some extent, but in chronic cases, its efficacy diminishes over time and may provide only temporary pain relief. The universal positive response to local anesthetic blocks prior to surgery should be considered when selecting candidates for TMR, but this alone is not predictive of long-term success. The prolonged duration of pain since the initial trauma in our patients may have led to central synaptic changes, perpetuating pain independently of peripheral nerve stimulation. This underscores the importance of early intervention. Prophylactic TMR performed closer to the time of injury might yield more effective results, potentially preventing the development of symptomatic neuromas, particularly in areas with high cortical representation like the volar digital nerves. Future research should focus on larger patient cohorts and longer follow-up periods to better understand the optimal timing and conditions for TMR. In conclusion, while TMR remains a viable option for managing painful neuromas, its application should be carefully considered, particularly in chronic cases. In such cases, the long-term results are not established due to a lack of reports in the literature. Considering our experience and literature findings, pain relief for painful neuromas of distal sensory nerves may be achievable but may not be definitive and could only provide short-term results, especially when a long time has passed since the injury. We suggest thinking about TMR and/or other procedures for treating painful neuromas soon after symptoms come up.

Patients should be fully informed of the potential for limited pain relief and the importance of early intervention to maximize the chances of success.

## Figures and Tables

**Table 1:** Type of trauma, nerves involved, and type of treatment before TMR.

Patient	Age	Date of Trauma	Type of Trauma	Nerves Involved	Type and Time of First Procedure	Revision Procedures
1	59	November 2017	Crush-avulsion injury at P2 of the right thumb	Volar collateral nerves of the thumb. Dorsal branches of the radial nerve	Revision of amputation in 2020	February 2021: Amputation of the stump and CCA of colletaral nerves May 2022: Median nerve decompression at the wrist and proximal forearm January 2023: Radial nerve decompression for Wartenberg’s Syndrome
2	70	January 2019	Crush injury of middle and ring fingers at P1 (left hand)	Collateral digital nerves of middle and ring fingers	Amputation of the ring finger at P1, amputation of the middle finger at P1 two months after trauma	November 2021: Neuroma resection (both collateral of III and radial of IV) and neurocap
3	36	July 2020	Motor hoe injury of the middle third of the right leg	Tibial nerve	Skin suture without exploration and nerve repair	September 2021: Tibial nerve reconstruction with sural graft

P2: second phalanx, P1: first phalanx.

**Table 2:** Timing of TMR and results according to NRS and DN4 scoring system.

Patient	Time Between Trauma and TMR (Months)	Type of Procedure	NRS/DN4 Before TMR	NRS/DN4 3 Months	NRS/DN4 6 Months	NRS/DN4 12 Months	NRS/DN4 Last Follow-Up	Time Without Pain (Months)	Follow-Up Time (Months)
1	65	April 2023: Superficial radial nerve to anterior interosseous nerve	NRS 8 DN4 8	NRS3 DN4 4	NRS 7 DN4 7	NRS 7 DN4 7	NRS 7 DN4 7	2	12
2	44	September 2022: Neuroma resection and TMR radial nerve of long finger on lumbricalis motor nerve, direct muscle neurotization ulnar nerve on lumbricalis, centro central union ring finger nerves	NRS 9 DN4 6	NRS 0 DN4 2	NRS 0 DN4 2	NRS 5 DN4 5	NRS 7 DN4 6	6	19
3	25	October 2023: Proximal neuroma resection and TMR on flexor digitorum longus	NRS 9 DN4 5	NRS 7 DN4 5	NRS 5 DN4 5	NRS 5 DN4 5	NRS 5 DN4 5	1	12

## Data Availability

The original contributions presented in this study are included in the article. Further inquiries can be directed to the corresponding author.
